# Somatic comorbidities and health related uncertainty among Swedish adolescents with ADHD

**DOI:** 10.3389/fpsyt.2025.1534280

**Published:** 2025-02-11

**Authors:** Sara Lundqvist, Sara Röjås, Kourosh Bador, Maria Råstam, Nóra Kerekes

**Affiliations:** ^1^ Department of Psychiatry and Neurochemistry, Sahlgrenska Academy, University of Gothenburg, Göteborg, Sweden; ^2^ Department of Child and Adolescent Psychiatri, Sahlgrenska University Hospital, Göteborg, Sweden; ^3^ Centre for Holistic Psychiatry Research (CHoPy), Mölndal, Sweden; ^4^ Department of Clinical Sciences, Faculty of Medicine, Lund University, Lund, Sweden; ^5^ Department of Health Sciences, University West, Trollhättan, Sweden

**Keywords:** adolescents, allergies, health-related uncertainty, attention-deficit/hyperactivity disorder (ADHD), gender, migraine, skin diseases

## Abstract

**Introduction:**

Attention-deficit/hyperactivity disorder (ADHD) frequently exists alongside psychiatric comorbidities. The coexistence of somatic diseases and ADHD constitutes a growing field of research.

**Method:**

This study aimed to examine the prevalence of common somatic diseases in adolescents with ADHD and compare them with those in adolescents without any psychiatric diagnoses. A cross-sectional study was conducted among a convenient sample of Swedish upper secondary school students aged 15 to 19 years. Data were collected using an electronic version of the “Mental and Somatic Health without borders” survey. In 2020, 1608 adolescents completed the survey and provided self-reports of their psychiatric and somatic diagnoses.

**Results:**

Among the sample of Swedish adolescents, 5.5% reported having ADHD diagnoses. These adolescents reported more somatic comorbidities than those without any psychiatric diagnosis (comparison group). The most common somatic comorbidities among those with ADHD were allergies (43.4%), asthma (24.7%), and skin diseases (16.7%), which prevalences were significantly higher than those reported by adolescents in the comparison group. However the effect sizes of these differences were negligible (allergies: p=0.002, Cramer’s V=0.08; asthma: p=0.041, Cramer’s V=0.06; skin diseases: p=0.007, Cramer’s V=0.08), raising questions about the practical implications of these findings. Allergies were significantly more common in both genders with ADHD (p=0.038, Cramer’s V=0.08 in women and p=0.038, Cramer’s V=0.09 in men). Additionally, men with ADHD showed a significant association with skin diseases (p=0.007, Cramer’s V=0.12) while women with ADHD were significantly more likely to have migraine (p=0.038, Cramer’s V=0.08). Notably, adolescents with ADHD reported significantly higher rates of uncertainty regarding the existence of diabetes, rheumatoid diseases, asthma (with negligible effect sizes), and thyroid diseases (with a small effect size) than those without any psychiatric diagnoses, suggesting heightened health related anxiety within this group.

**Conclusions:**

Adolescents with ADHD reported more somatic comorbidities and greater uncertainty about the potential presence of additional somatic conditions compared to adolescents without psychiatric diagnoses. These findings highlight the importance of addressing both health literacy and access to healthcare. By focusing on this group, we cannot only improve their ability to understand and navigate the healthcare system but also strengthen their confidence in managing their own health.

## 1 Introduction

Attention-deficit/hyperactivity disorder (ADHD) is a neurodevelopmental disorder that typically begins in childhood and is characterized by symptoms of hyperactivity, impulsivity, and/or inattention (1). The estimated worldwide prevalence of ADHD in adolescents aged 12 to 18 is 5.6% (2). In Sweden, the prevalence of ADHD among children and adolescents aged 10 to 17 was 4.5% in girls and nearly 9% in boys in 2020, according to the National Board of Health and Welfare (3). ADHD is believed to arise from a combination of genetic and environmental risk factors. A substantial body of research supports the conclusion that no single risk factor is sufficient to cause ADHD. Additionally, individuals with ADHD are at an elevated risk of developing co-occurring psychiatric and somatic conditions (4). In adults with ADHD, increased genetic vulnerability is associated with a modestly increased risk of various somatic health problems later in life as well as increased risks of multiple somatic comorbidities and autoimmune diseases (5–7).

Adolescents with ADHD also exhibit higher rates of somatic diseases, though these associations show considerable heterogeneity (8). For instance, a 2017 systematic review and meta-analysis that involved more than 61,000 children found that individuals with ADHD were more likely to have allergic rhinitis, asthma, atopic dermatitis, and allergic conjunctivitis than those without ADHD (9). One Swedish study revealed the connections between various chronic conditions, such as asthma and allergy, and ADHD in adolescents, and another illustrated the connection between childhood-onset type 1 diabetes and various developmental disabilities (10, 11). Additionally, a nationally representative sample of US children and adolescents found significant positive associations between ADHD and food allergies, respiratory allergies, and skin allergies (12). Furthermore, a national Danish register study that included nearly one million people suggested that a personal history of autoimmune disease was associated with an increased risk of ADHD (13). Conversely, a 2024 systematic review and meta-analysis, which included over 250,000 children across 20 countries, failed to replicate previously reported the significant associations between ADHD and asthma and eczema. This highlights the variability of findings across different studies (8) and underscores the importance of reporting effect sizes in studies to convey practical relevance, rather than focusing solely on statistical significance.

The frequent co-occurrence of ADHD and various inflammatory and autoimmune disorders indicates the presence of underlying mechanisms that are still being investigated (14). These mechanisms may involve altered immune responses and shared genetic and environmental factors, although the precise relationships have not been fully elucidated (13, 15).

The presence of comorbid somatic disorders in adolescents with ADHD further impairs these individuals’ functioning and reduces their quality of life. Therefore, raising awareness among healthcare providers about the high prevalence of somatic comorbidities in individuals with ADHD is crucial.

The present study aimed to describe the self-reported prevalence of common somatic diseases among adolescents with ADHD from the general Swedish population and compare these rates with those among adolescents who reported no existing psychiatric conditions. Additionally, this study examines and discusses these adolescents’ uncertainty and anxiousness regarding the existence of their own somatic comorbidities.

## 2 Methods

### 2.1 Study design and procedure

This international study, initiated in Sweden, adopted a cross-sectional design. The data for this study were collected using convenience sampling through virtual means, via an electronic survey known as the “Mental and Somatic Health without borders” (MeSHe) survey. The data collection in Sweden commenced in September 2020, and the substantial majority (82.47%) of the information was gathered during the December 2020 Christmas holiday period. Convenience sampling was utilized due to the constraints of the COVID-19 pandemic, which restricted access to traditional probability-based sampling methods. This approach allowed for efficient data collection across all 21 counties in Sweden, capturing diverse responses within the constraints of the study period.

Within the MeSHe survey, adolescents completed various validated questionnaires regarding their mental and physical well-being, risk behaviors, general affective states, and personality traits. For a comprehensive overview of all the instruments in the MeSHe survey, please refer to https://meshe.se/. The data for this particular study were based on the participants’ responses to the MeSHe health questionnaire.

### 2.2 Study population

Data from a total of 1608 respondents fulfilled the national ethical committee’s requirement regarding the determined age span of participation between 15 and 19 years old. Of these 1608 respondents, 1 person did not specify gender; therefore, the present study utilized data from 1607 Swedish adolescents. The analyses included responses from 630 adolescent men, 962 adolescent women, and 15 adolescents of other gender. The respondents’ average age was 17.15 years (standard deviation=0.88, median=17).

### 2.3 Instrument: the MeSHe health questionnaire

The MeSHe health questionnaire comprised queries wherein adolescents indicated the presence of specified somatic diseases (with response options of “yes,” “no,” and “I do not know”). The list of somatic diseases included allergies, asthma, celiac disease, diabetes, migraine, rheumatoid diseases, skin diseases, and thyroid diseases. The MeSHe health questionnaire has been validated for use in adolescent populations (16, 17), including a study conducted within the Swedish adolescent demographic (17). The questionnaire has demonstrated acceptable test-retest reliability (16).

In addition to reporting somatic diseases, the adolescents were asked to indicate whether they had received a psychiatric diagnosis from a healthcare professional (response options: ‘yes’ or ‘no’). Those who responded affirmatively could further specify the diagnoses in free-text format. For the present study, the psychiatric diagnoses indicated in the free text were categorized into 10 diagnostic groups: attention deficit disorder and attention-deficit/hyperactivity disorder (ADHD), autism spectrum disorder, depression, anxiety, post-traumatic stress disorder, obsessive-compulsive disorder, bipolar disorder, eating disorders, other disorders, and non-specific disorders). This categorization was carried out by a specialist in child and adolescent psychiatry (SL) in collaboration with a resident in child and adolescent psychiatry (SR). Individuals who indicated diagnoses of attention deficit disorder and ADHD are collectively referred to as “adolescents with ADHD” or “the ADHD group” in this study. Adolescents who indicated no existing psychiatric diagnoses constituted the comparison group.

### 2.4 Statistical analysis

All analyses were conducted using IBM SPSS Statistics, version 28. Descriptive statistics were employed, with frequencies presented as percentages (%). The chi-squared test was used to compare the prevalence of somatic diseases between the groups, and odds ratios and 95% confidence intervals were defined. Cramer’s V was used to indicate effect sizes (small [0.10–0.29] or medium [>0.30]). The non-parametric Mann-Whitney U test was employed to detect significant differences in the number of somatic diseases between adolescents with ADHD and those without any psychiatric disorders. In this analysis, the effect size was calculated using η² = Z²/(N-1), with a threshold of 0.01 indicating a small effect size. The level of significance was set at p<0.05.

## 3 Results

Of the 1607 respondents, 14 did not respond to the question about the existence of any psychiatric diagnoses, resulting in an internal attrition rate of 0.9%. The mean age of these 14 adolescents was 17.14 years. Among them, 35.6% (n = 5) identified as male, 50% (n = 7) as female, and 14.2% (n = 2) as identifying with neither gender. One respondent (7%) reported the presence of a somatic disease.

Among the remaining 1593 respondents, 312 (19.6%) reported having at least one psychiatric diagnosis. Of those individuals, 87 (27.9% of those who indicated having at least one psychiatric diagnosis) specifically indicated ADHD, either with or without co-existing psychiatric disorders, in their free-text responses. The gender distribution of those 87 adolescents was as follows: 46 were boys/men (53% of the ADHD group; 8.2% of all male adolescents), 39 were girls/women (45% of the ADHD group; 4.9% of all female adolescents), and two were of non-binary gender (2% of the ADHD group; 22.2% of all non-binary adolescents). In the study population of 1593 Swedish adolescents, the self-reported prevalence of ADHD was 5.46% (n=87); 1281 (80%) reported not having any psychiatric diagnoses, therefore constituting the comparison group. Of the individuals in this comparison group, 59.3% identified as girls/women, 40.1% identified as boys/men, and 0.5% identified as non-binary.

The study assessed the prevalence of various somatic diseases, including allergies, asthma, celiac disease, diabetes, migraine, rheumatoid diseases, skin diseases, and thyroid diseases, within the study population. Adolescents with ADHD had approximately twice the odds of having asthma, allergies, and skin diseases as the comparison group (**Table 1**). Although the prevalence of these somatic diseases differed significantly between the groups, the effect sizes were negligible. The prevalence of rheumatoid diseases was similar in both groups. The comparison group exhibited higher rates of diabetes and thyroid diseases than the ADHD group; however, these differences were not statistically significant (**Table 1**).

**Table 1:** The prevalence of somatic diseases in adolescents with ADHD and in those without psychiatric diagnoses.

	Comparison group (%)	ADHD group (%)	*p*	Cramer’s V	OR	95% CI
Allergies	27.8	43.4	0.002	0.08	1.99	1.27–3.12
Asthma	16.0	24.7	0.041	0.06	1.72	1.02–2.92
Celiac disease	1.6	2.4	0.57	-	-	
Diabetes	0.6	0.0	0.46	–		
Migraine	7.5	10.8	0.27	-	-	
Rheumatoid diseases	1.1	1.3	0.87	–	–	
Skin diseases	8.1	16.7	0.007	0.08	2.28	1.24–4.20
Thyroid diseases	0.6	0.0	0.48	–	–	

OR, odds ratio; CI, confidence interval.

When the analyses were repeated separately for adolescent man and women, similar patterns were observed. Among adolescent women with ADHD, the prevalence of asthma, rheumatoid disease, and skin diseases was substantially higher but did not reach statistical significance. Allergies (27.5% in the comparison group vs. 43.2% in the ADHD group) and migraine (7% in the comparison group vs. 16.2% in the ADHD group) showed statistically significant increases (p = 0.038 for both conditions). The odds ratios for these conditions indicated approximately doubled odds (allergies: OR = 2.20, 95% CI = 1.03–3.93; migraine: OR = 2.56, 95% CI = 1.02–6.43). However, the significant differences did not reach the threshold for a small effect size (Cramer’s V = 0.08).

For adolescent man with ADHD, the prevalence of asthma and celiac disease was notably higher but did not reach statistical significance. Significant differences were observed in the prevalence of allergies (28.2% in the comparison group vs. 43.2% in the ADHD group; p = 0.038) and skin diseases (6.5% in the comparison group vs. 14.7% in the ADHD group; p = 0.007). The effect size for allergies approached the threshold for a small effect size (Cramer’s V = 0.09), while the effect size for skin diseases met the criterion for a small effect (Cramer’s V = 0.116, OR = 3.02, 95% CI = 1.30–7.02).

A significant portion of the study population reported uncertainty regarding whether they had some of these somatic diseases, as indicated in **Table 2**. Notably, the adolescents with ADHD indicated significantly higher rates of uncertainty regarding the existence of asthma (with a negligible effect size), diabetes, rheumatoid diseases, and thyroid diseases (with small effect sizes) than the comparison group. Adolescents with ADHD were nearly twice as likely to be unsure of their diabetes status, more than twice as likely to be unsure of their asthma status, nearly six times as likely to be unsure about thyroid diseases, and more than seven times as likely to be unsure of the presence of rheumatoid diseases (**Table 2**).

**Table 2:** The percentages of adolescents reporting uncertainty regarding specific somatic diagnoses.

	COMPARISON GROUP		ADHD GROUP		*P*	CRAMER’S V	OR	95% CI
	n	I don’t know (%)	n	I don’t know (%)				
Allergies	1271	5.0	86	3.5	0.54	–	–	
Asthma	1271	3.0	87	6.9	0.046	0.05	2.40	0.99–5.85
Celiac disease	1273	1.0	86	2.3	0.26	–	–	
Diabetes	1270	0.2	87	2.3	<0.001	0.10	14.92	2.08–107.21
Migraine	1262	5.1	85	2.4	0.26	–	–	
Rheumatoid diseases	1270	2.1	87	13.8	<0.001	0.17	7.37	3.59–15.16
Skin diseases	1260	2.5	86	2.3	0.90	–	–	
Thyroid diseases	1257	1.3	84	7.1	<0.001	0.11	5.97	2.27–15.67

OR, odds ratio; CI, confidence interval.

**Figure 1:**
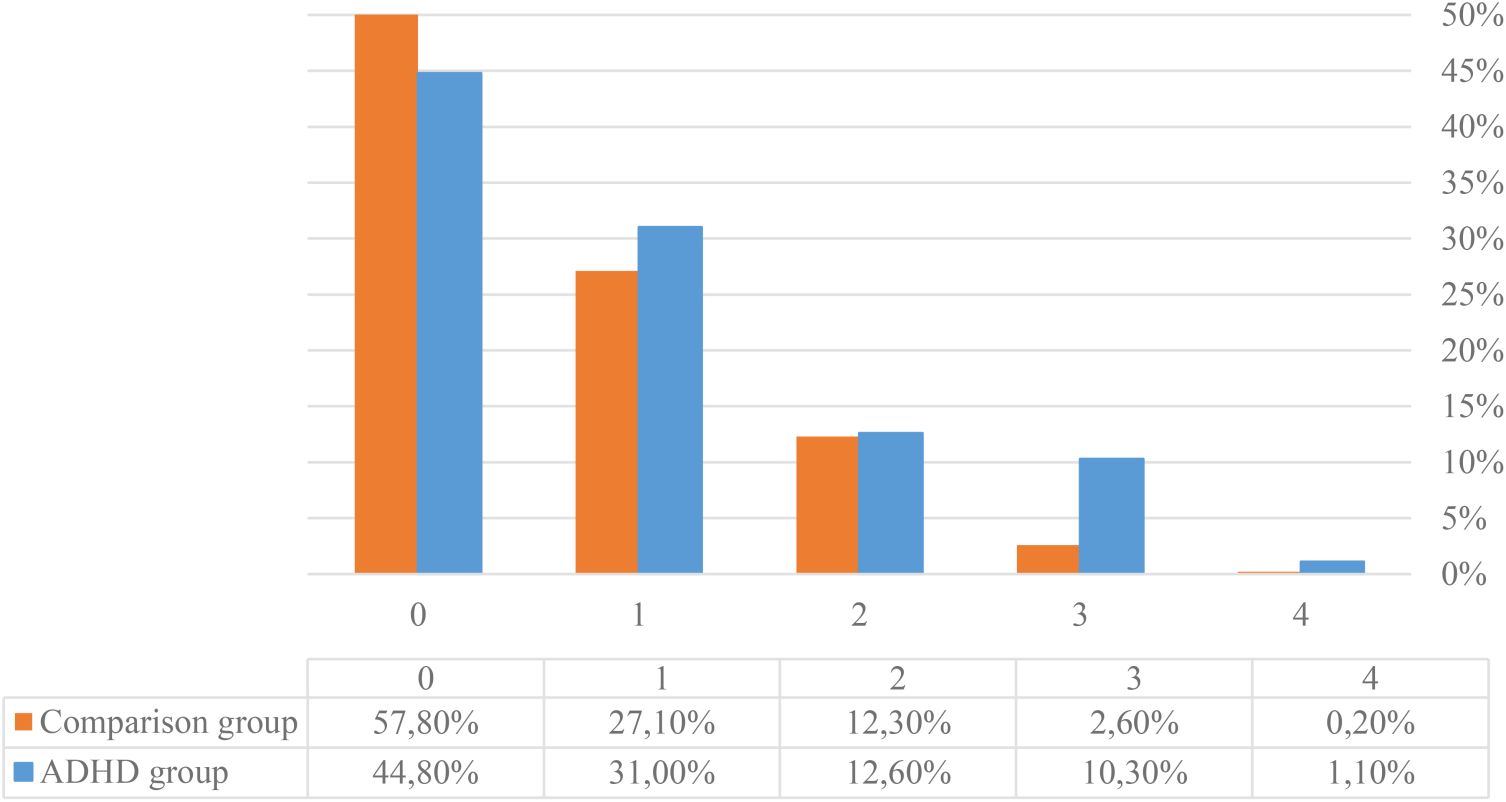
The percentages of respondents within the groups of adolescents with ADHD and those without any psychiatric diagnoses reporting none or up to four coexisting somatic diseases.

There was a significant difference in the number of somatic diseases between the two groups (p=0.005). The adolescents with ADHD reported significantly more somatic diseases than those without any psychiatric diagnoses, but this difference also had a very small effect size (η^2^ = 0.006) (**Figure 1**).

## 4 Discussion

The present study indicates greater vulnerability in the physical health and heightened anxiety of adolescents with ADHD than in that of adolescents without any psychiatric diagnoses based on self-reported data from the general population.

Adolescents with ADHD reported significantly more coexisting somatic conditions. These results support previous findings that ADHD and physical illness are closely connected, both in adolescents and adults (4, 8, 18, 19). However, it is essential to consider the limitations of relying solely on statistical significance when interpreting these associations. Researchers often use statistical significance to determine whether an observed difference is likely to be due to chance or if it reflects a true difference in the populations being studied. Yet, statistical significance alone does not necessarily indicate clinical or practical relevance. The inclusion of effect sizes offers a clearer understanding of the practical implications of these findings. An effect size measures the magnitude of that difference, providing insight into whether a statistically significant difference is meaningful in real-world contexts. In the present study, while the differences in the prevalence of allergies, asthma, and skin diseases between the ADHD group and the comparison group were statistically significant the effect sizes were negligible. Even in the gender-specific analyses, we found that only skin diseases in adolescent man with ADHD and without any psychiatric disorders differed significantly, reaching a small effect size. Allergies in both genders and migraine in women with ADHD were significantly more prevalent compared to those without any psychiatric conditions; however, these significant differences exhibited negligible effect sizes. This suggests that, although differences exist, their impact on clinical practice or daily life may be minimal. These findings align with a recent systematic review and meta-analysis that highlighted the substantial heterogeneity in the association between ADHD and comorbid somatic diseases across different study populations (8).

In our study, the prevalence of other inflammatory conditions, such as rheumatoid diseases and thyroid diseases, did not significantly differ between adolescents with ADHD and those without any psychiatric diagnoses, but was increased, mainly in adolescent girls. However, noteworthy proportions of adolescents with ADHD reported uncertainty regarding whether they had these diseases. Some of these significant differences had small effect size, indicating small but practically relevant implications.

There are several potential explanations for this uncertainty. This uncertainty may suggest the possible presence of related symptoms in the absence of official diagnoses. These uncertain young people did not give an affirmative answer, but neither did they say no. This uncertainty can be interpreted as part of the uncertainty and stressed anxiety that characterizes young people with ADHD. It is common with somatic complaints that do not add up to a specified somatic disorder. These findings may be consistent with well documented increased anxiety in ADHD in adolescence, often accompanied with somatic complaints (20, 21). ADHD in young people has repeatedly been associated with anxiety and even anxiety disorders, problems that in turn have been linked to concerns about the body and symptoms from the body. Not infrequently the somatic symptoms have changed from time to time and, even in cases with substantial problems no specific diagnosis has been made (20, 21).

From another perspective, to properly answer the questions in the survey, one must be knowledgeable about the relevant illnesses. Rheumatoid and thyroid diseases are relatively uncommon diseases with which one cannot confidently expect adolescents to be familiar. Previous studies have shown that adolescents with ADHD have more complaints about their health (21). Therefore the high rates of uncertainty observed in the present study may involve undiagnosed illnesses. This highlights the importance of improving health literacy and awareness among adolescents, particularly those with ADHD.

Overall, our findings align with the results of previous studies that have also reported elevated rates of inflammatory diseases among children and adolescents with ADHD (8–13, 19). Conditions such as allergies, asthma, skin diseases, rheumatoid diseases, and thyroid diseases are all associated with childhood onset and have an inflammatory nature. There is strong evidence that indicates immune dysregulation and an increased pro-inflammatory state in individuals with various psychiatric disorders (22–24). However, this does not imply that the exact causal relationship can currently be elucidated.

Specifically, the evidence that supports the connection between inflammation and ADHD is growing, primarily deriving from observational studies that indicate the frequent co-occurrence of ADHD and inflammatory and autoimmune conditions (such as the present study), genetic research, and clinical investigations that assess serum inflammatory markers (14). Substantial evidence supports the hypothesis that ADHD is linked to inflammation and immune-related processes. It is postulated that inflammatory mechanisms play a role in the pathophysiology of various neuropsychiatric disorders through mechanisms such as neuronal damage, glial activation, increased oxidative stress, diminished neurotrophic support, and altered neurotransmitter metabolism (22, 25, 26).

The gender-specific analyses in this study provide valuable insights into the associations between ADHD and somatic diseases in adolescents, revealing distinct patterns of comorbid conditions between man and women. Allergies were significantly more prevalent in both genders with ADHD, while asthma showed an increased prevalence in both groups but did not reach statistical significance. Skin diseases were also more commonly reported in both genders; however, the increase was statistically significant only in man with ADHD, where it reached a small effect size. Migraine was significantly more prevalent in adolescent women with ADHD, while rheumatoid diseases showed a higher prevalence in girls but did not achieve statistical significance. In adolescent man with ADHD, celiac disease was more prevalent but also fell short of statistical significance.

These findings align with prior research suggesting gender differences in the prevalence of certain somatic conditions. For example, conditions such as migraine, rheumatoid diseases, and thyroid disorders are known to be slightly overrepresented among females compared to males (27–29).

Our results are in line with those of previous national and international research and reinforce the need for integrated care for adolescents with psychiatric and somatic comorbidities. Childhood disease places high demands on parents and, later, on adolescents. Inflammatory and autoimmune conditions require careful treatment. This treatment is sometimes temporary and sometimes continuous, but it must nevertheless be managed in a specific way. Patients and their parents must follow treatment routines, understand the consequences of neglecting treatment, and keep track of timing and medication dosages. These particular skills are often challenging for children and adolescents with ADHD. The growing body of evidence regarding somatic difficulties in children and adolescents with ADHD prompts us to contemplate the design of treatment guidelines for somatic conditions that are tailored to the unique needs of adolescents with ADHD.

## 5 Conclusion

While the associations between common somatic conditions and ADHD show great variation in strength, their (partially gender-specific) co-existence is reinforced in the present study. In addition to the increased number of somatic complaints reported by adolescents with ADHD, they also exhibited heightened anxiety related to thoughts or symptoms of undiagnosed somatic conditions. Recognizing and addressing comorbid somatic conditions and elevated health-related anxiety in adolescents with ADHD is crucial for enhancing these individuals’ overall health and quality of life. Future research should focus on elucidating the underlying mechanisms of these associations and developing targeted interventions to mitigate the health burdens that this vulnerable population faces. This will ensure more effective management and better health outcomes for adolescents with ADHD, ultimately improving their quality of life.

Collaboration between child and adolescent psychiatry practitioners, primary care physicians, and even teachers to enhance adolescents’ health literacy is essential for the development of effective support and treatment programs for conditions prevalent in this population.

## 6 Strengths and limitations

One notable strength of the current study is the considerably large number of young Swedish adolescents from the general population who responded to the health questionnaire, with responses collected from every county in Sweden. However, it is important to note that the study population is not representative due to the use of a non-probabilistic sampling method. While this approach allowed for timely and efficient data collection during the COVID-19 pandemic, it introduces potential biases, such as self-selection bias, as individuals with heightened health awareness or existing concerns may have been more inclined to participate. Additionally, the study exclusively utilized anonymous self-reports, precluding confirmation of the respondents’ answers regarding diagnosed illnesses through medical records. While using self-reported data presents a limitation of this study, it also serves as a strength, allowing us to capture participants’ uncertainty and heightened anxiety regarding their somatic health—information that is often absent in clinical registers.

The MeSHe health questionnaire, utilized in this study, has been validated for adolescent populations and has been applied in previous research within Sweden (17). Its demonstrated test-retest reliability (16) supports its consistency and suitability for assessing health perceptions among adolescents. While the questionnaire has been validated for use in Sweden and similar contexts, we recognize the importance of further validation efforts across diverse populations to enhance its generalizability and methodological rigor in future studies.

Reflecting on the low internal attrition rate (0.9%), it is notable that among those who did not respond to the question about psychiatric diagnoses, the prevalence of individuals who did not identify as man or women was higher. However, their mean age did not differ from the rest of the study population. Fewer than half of these individuals responded to the question regarding somatic conditions, and among those who did (n = 6), only one (17%) reported having a somatic disease. This represents a lower prevalence compared to both the comparison and the ADHD groups. These findings may suggest a potential gender-related bias, as adolescents who do not identify with binary genders could experience greater challenges in responding to health-related questions. Previous research highlights the unique mental and somatic health challenges faced by non-binary adolescents, which may contribute to differences in survey engagement and responses (30). This warrants further investigation to better understand how non-binary adolescents engage with health-related surveys and whether tailored approaches are needed to ensure inclusivity and accurate data representation.

Even the results of our gender-specific analyses underscore the importance of considering gender differences in future research on ADHD and somatic comorbidities. Further studies with larger sample sizes for subgroup analyses are needed to confirm and refine these observations, ensuring a deeper understanding of the interplay between ADHD, somatic health, and gender.

It is also worth acknowledging that the data used in the present study were collected during the COVID-19 pandemic. According to a study that investigated the impact of the pandemic on this study population, Swedish adolescents demonstrated high resilience and experienced minimal or negligible effects on their overall health (31). Therefore, the impact of the COVID-19 pandemic on the reported prevalence rates in this study is likely negligible.

## Data Availability

The raw data supporting the conclusions of this article will be made available by the authors, without undue reservation.
